# Dual Signal Enhancement by Magnetic Separation and Split Aptamer for Ultrasensitive T-2 Toxin Detection

**DOI:** 10.3390/molecules30132853

**Published:** 2025-07-04

**Authors:** Ziyi Yan, Ping Zhu, Chaoyi Zhou, Dezhao Kong, Hua Ye

**Affiliations:** 1 School of Grain Science and Technology, Jiangsu University of Science and Technology, Zhenjiang 212003, China; yanziyi20@hotmail.com (Z.Y.); zhupiiing0605@163.com (P.Z.); 242212188423@stu.just.edu.cn (C.Z.); kdz1011@just.edu.cn (D.K.); 2 Jiangsu Provincial Engineering Research Center of Grain Bioprocessing, Jiangsu University of Science and Technology, Zhenjiang 212003, China

**Keywords:** split aptamer, biosensor, magnetic graphene oxide, T-2 toxin

## Abstract

T-2 toxin, a type A trichothecene mycotoxin produced by *Fusarium* species, is widely present in cereals and their processed products, posing a significant contaminant in food safety. To address the food safety challenges caused by this toxin, we established a dual signal enhancement by magnetic separation and split aptamer for ultrasensitive T-2 toxin detection. In this method, the introduction of magnetic graphene oxide (MGO) enhanced signal and increased sensitivity by reducing background interference. The shortened split aptamer reduces non-specific binding to MGO via decreased steric hindrance, thereby facilitating rapid target-induced dissociation and signal generation. A FAM fluorophore-labeled split aptamer probe FAM-SpA1-1 was quenched by MGO. While the fluorescence intensity remained nearly unchanged when the unlabeled split aptamer probe SpA1-2 was introduced alone, a significant fluorescence recovery was observed upon simultaneous addition of SpA1-2 and T-2 toxin. This recovery resulted from the cooperative binding of SpA1-1 and SpA1-2 to T-2 toxin, which distanced the FAM-SpA1-1 probe from MGO. Therefore, the proposed biosensor demonstrated excellent stability, reproducibility, and specificity, with a linear response range of 10–500 pM and a limit of detection (LOD) of 0.83 pM. Satisfactory recovery rates were achieved in spiked wheat (86.0–114.2%) and beer (112.0–129.6%) samples, highlighting the biosensor’s potential for practical applications in real-sample detection. This study establishes the T-2 toxin split aptamer and demonstrates a novel dual-signal enhancement paradigm that pushes the sensitivity frontier of aptamer-based mycotoxin sensors.

## 1. Introduction

T-2 toxin is usually a type A trichothecene mycotoxin produced by *Fusarium species* such as *Fusarium pearsporium*, *Fusarium trilinea*. Its characteristic structure features a tetracyclic 12, 13-epoxytrichothec-9-ene core (common to all trichothecenes), with specific hydrophobic groups (isovaleryl ester group at the C-8 position and hydroxyl groups at C-4 and C-15) (Figure S1). It accumulates in the human body and can readily induce neurotoxicity, immunotoxicity, and reproductive toxicity, posing significant risks to human health [1,2,3]. Improper storage of cereal products such as wheat and corn is susceptible to mildew and growth of *Fusarium* and poses a risk of T-2 toxin contamination [4,5]. Therefore, developing accurate and precise detection methods for T-2 toxin is critical to safeguarding food safety and public health. Traditional methods, including chromatographic techniques and immunoassays, have demonstrated utility in laboratory settings. While chromatographic methods exhibit superior sensitivity, they suffer from expensive and sophisticated instruments, tedious sample pretreatment process and professional personnel, technically demanding protocols, and high operational costs, fundamentally limiting field applicability [6,7]. Conversely, immunoassays possess quick analysis speed and excellent selectivity. However, antibody preparation is time-consuming and costly; pre-labeling them with reporters can affect their properties and non-specific reactions often lead to false-positive results [8,9,10]. Indirect Competitive Enzyme-Linked Immunosorbent Assay (ic-ELISA) (Table 1) is an indirect competitive immunoassay based on antigen–antibody specific reactions, which is complex and time-consuming. While iSPR biosensors improve detection performance, their manufacturing and operating costs can be high, limiting their widespread use in resource-constrained environments. SPR sensors are used for real-time, label-free monitoring of biomolecular interactions. However, sensitivity is limited, especially when detecting targets at low concentrations, and is more susceptible to interference from external noise. Portable microfluidic devices may be affected by environmental factors (such as temperature and humidity) in practical applications, resulting in a decrease in the stability of detection results. Consequently, it is urgent to develop a simple, sensitive, rapid T-2 toxin detection method to meet the needs of rapid detection and food safety supervision.

In recent years, aptamers—often termed “chemical antibodies”—have emerged as versatile biorecognition elements because they can fold into specific three-dimensional structures and bind specifically to targets [11]. Because of its good stability, excellent economy, convenient modification, and high sensitivity [12,13], it is widely used for probes to construct colorimetric [14,15,16], fluorescent [17,18], electrochemical [19,20], and other types of biosensor. For example, Deng et al. developed a real-time detection method for T-2 toxin utilizing target-responsive DNA hydrogels coupled with potassium iodide starch test strips. This approach was successfully applied to the detection of T-2 toxin in real corn samples. Under optimized conditions, the method reported a broad detection range from 10 ng/mL to 10 mg/mL and claimed a low limit of detection. This colorimetric distance dual-signal detection method has significant advantages, such as simple operation, clear signal interpretation, and strong practicability [21]. However, a single aptamer molecule with redundant structure can be affected by interferences and non-specific effects in complex food matrices, leading to false-positive results [22]. Fortunately, split aptamers may be effective in avoiding this and in increasing the reliability of the test results. For example, Wang et al. split the stem-loop-structured bisphenol A aptamer into two fragments (SPA-a and SPA-b) and developed an enzyme-free amplification detection system through dual-terminal labeling. The results showed that the split dual-labeled probe exhibited significantly enhanced performance compared to the intact single-labeled probe, achieving a detection limit as low as 0.89 ng/mL. Notably, the detection sensitivity of the dual-labeled probe was 24-fold higher than that of its single-labeled counterpart [23]. Qi et al. developed a split aptamer-based 3D-DNA walker biosensor for ultra-sensitive and specific detection of 17β-estradiol (E2) in food samples, achieving a high signal-to-background ratio. Upon target-induced structural recombination of two split probes (STWS-a and STWS-b), the formed walking strand (STWS) activated E6-DNAzyme, driving 3D-DNA walker for signal amplification. The biosensor exhibited a good linear range of 1 pM to 50 nM with a remarkably low LOD of 0.28 pM, along with excellent sensitivity (recoveries of 95.6–106.5%) in complex foods. The split aptamer design—featuring short, simple strands that require both for target binding—enhanced detection reliability by preventing nonspecific interactions [24]. In previous work, our research group rationally designed and obtained a pair of split aptamer probes target to T-2 toxin. We elucidated their synergistic recognition mechanism and subsequently developed a dual-mode fluorescence/fluorescence polarization detection method for T-2 toxin. The results showed an excellent detection property of the aptasensor, indicating a linear range of 0.1–20 nM and 0.2–50 nM with detection limit of 0.1 nM, 0.12 nM for fluorescence and fluorescence polarization signals, respectively [25]. The binding affinity of the optimized split aptamer was also quantified via a graphene oxide-based fluorescence quenching assay. The measured dissociation constant (Kd = 19.07 ± 1.67 nM) demonstrates that the split aptamer maintains favorable binding affinity compared to the original T40 aptamer (Kd = 61 nM [26]). This significant improvement in target recognition capability directly contributes to higher detection sensitivity in T-2 toxin analysis. Compared to graphene oxide, magnetic graphene oxide (MGO) demonstrates superior analytical performance through its unique combination of fluorescence quenching capability and superparamagnetic properties. These characteristics enable effective background interference elimination and magnetic separation enrichment, thereby enhancing detection sensitivity. For example, the MGO system effectively suppressed background signals and quenched upconversion nanoparticle (UCNP) fluorescence, achieving an ultralow detection limit of 7.3 pM [27]. This principle has also been successfully extended to zearalenone (ZEN) detection using CdTe quantum dots, where MGO implementation reduced the limit of detection to 2.9 mg/L through optimized fluorescence quenching [28].

**Table 1:** Limit of detection comparison across different T-2 toxin assays.

Method	LOD (ng mL^−1^)	References
Fluorescent aptamer sensors	0.387 ng/mL (0.83 pM)	This study
iSPR biosensors	1.2 ng/mL	[29]
SPR sensor	0.57 ng/mL	[30]
ic-ELISA	0.827 ng/mL	[31]
Portable microfluidic devices	1.3 ng/mL	[32]

Despite advances in mycotoxin detection, existing methods face dual limitations in achieving sensitivity while resisting matrix interference in complex foods. To bridge this gap, we developed a dual-signal enhancement strategy integrating (1) MGO for background suppression via simultaneous separation and quenching; (2) Split aptamers leveraging cooperative target binding for accelerated signal generation. This method demonstrates excellent sensitivity, stability, and reproducibility while maintaining operational simplicity characteristics that make it particularly suitable for field-deployable rapid detection applications with significant practical potential.

## 2. Results and Discussion

### 2.1. Detection Principle

**Figure 1:**
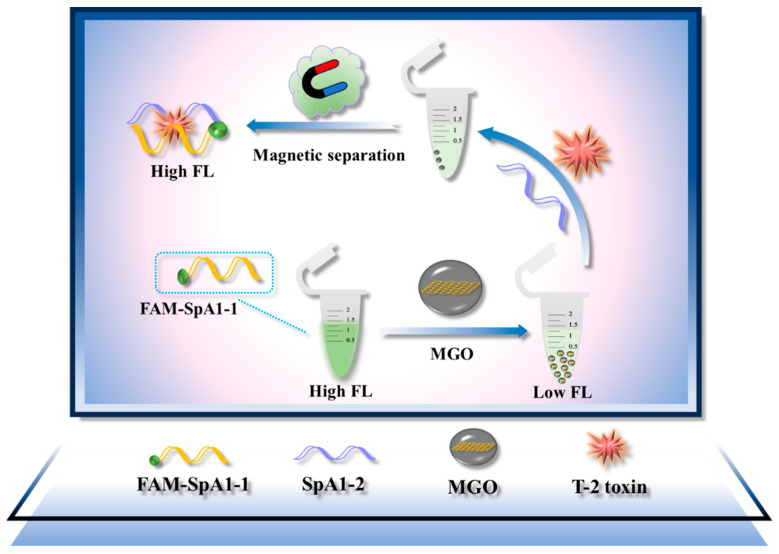
Schematic diagram of fluorescence detection of T-2 toxin based on split aptamer and MGO.

The split site was selected based on rigorous optimization in our previous study [25], which demonstrated that this configuration maximizes T-2 toxin binding efficiency while minimizing background signal in reassembled complexes. The working principle of the magnetically modulated split aptamer biosensor is illustrated in Figure 1. Firstly, MGO nanosheets were functionalized with SpA1-1 probes via p-p stacking interactions forming stable MGO/SpA1-1complex. The intrinsic fluorescence quenching capability of MGO effectively suppressed the fluorescence signal of fluorescein-labeled SpA1-1, establishing a baseline low-fluorescence state (F_0_). Upon introduction of T-2 toxin and SpA1-2, SpA1-1 was dissociated from MGO and self-assembled with SpA1-2 to form a specific three-dimensional structure by synergistically binding to T-2 toxin to form a SpA1-1/SpA1-2/T-2 ternary complex. Then MGO was removed by magnetic separation, and the fluorescence intensity of the supernatant was restored and measured. When SpA1-2 probe was introduced in the absence of T-2 toxin, the fluorescence intensity of the supernatant remains basically unchanged.

### 2.2. Optimization of Detection Conditions

**Figure 2:**
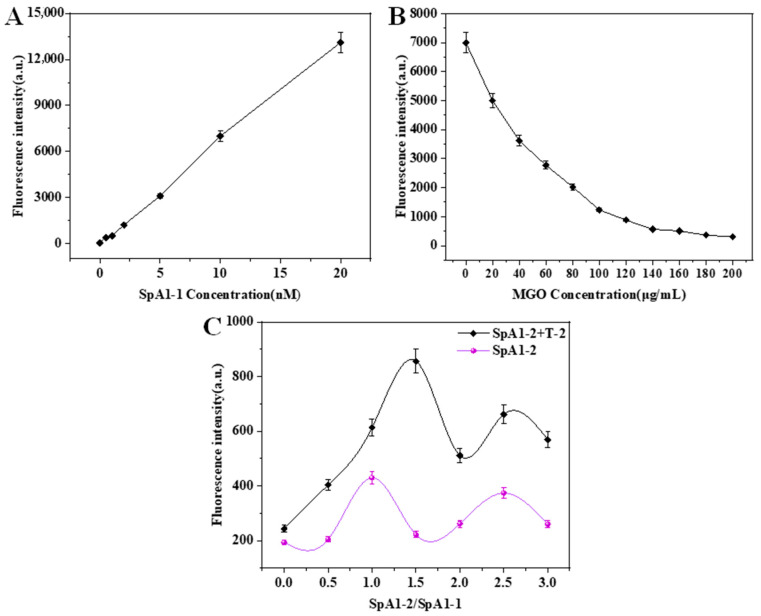
Optimization of detection concentrations: (**A**) FAM labeled-SpA1-1 concentration (0–20 nM); (**B**) MGO concentration (0–200 μg/mL); (**C**) Molar ratio of SpA1-2 and SpA1-1 (0:1, 0.5:1, 1:1, 1.5:1, 2:1, 2.5:1, 3:1).

To minimize systemic interference and enhance the detection performance of the split aptamer biosensor, the concentrations of FAM-SpA1-1 and MGO nanosheets, the molar ratio of SpA1-2 and FAM-SpA1-1, and the detection time were systematically optimized, and the results are shown in Figure 2. As can be seen in Figure 2A, the fluorescence intensity of the SpA1-1 system demonstrates a linear concentration-dependent response within the 0–20 nM range. Quantitative analysis reveals a fluorescence intensity of 13,000 at 20 nM. However, the fluorescence intensity still reaches about 7500 at 10 nM concentration. Considering cost-effectiveness, 10 nM was selected as the optimal working concentration for SpA1-1. Figure 2B shows the effects of different MGO concentrations on the fluorescence intensity of the 10 nM SpA1-1 system. Notably, the fluorescence intensity of the system gradually decreases with the increase in MGO concentration. When the MGO concentration increases above 100 μg/mL, the quenching efficiency reaches saturation, stabilizing the fluorescence intensity around 500. Therefore, the optimal concentration of MGO was determined to be 140 μg/mL, which can achieve 92% quenching efficiency.

Split aptamers are two strands that cooperatively bind T-2 toxin through structure assembly. To establish the optimal working ratio, the effects of molar ratio (SpA1-2/SpA1-1) on the fluorescence intensity were evaluated (Figure 2C). In T-2 toxin-containing systems (black curve), as the molar ratio increases, the fluorescence intensity initially increased proportionally up to 1.5, followed by a reduction at higher ratios (2 to 3). This suggests competitive inhibition from excess SpA1-2, potentially through non-specific strand displacement or steric interference with T-2 toxin binding. In T-2 toxin-free conditions (purple curve), fluorescence intensity remained stable except at 1:1 ratio, where a minor signal elevation indicated limited spontaneous assembly capability. It confirmed the essential cooperative role of SpA1-2 in both aptamer dissociation from MGO and subsequent recognition. These findings demonstrate that the optimal molar ratio (SpA1-2: SpA1-1) is 1.5.

**Figure 3:**
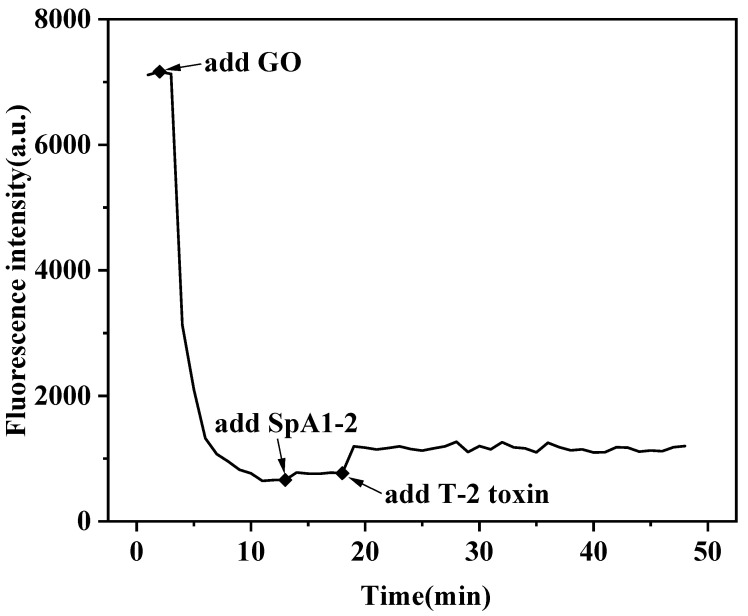
Fluorescence kinetic studies for detection time optimization following separate introduction of graphene oxide (GO) nanosheets, SpA1-2, and T-2 toxin into the SpA1-1 system.

The real-time fluorescence kinetic experiments were conducted to establish temporal parameters governing detection reliability and the result was shown in Figure 3. The FAM-SpA1-1 system exhibited three distinct kinetic stages after the addition of MGO, SpA1-2, and T-2 toxin. As can be seen, the fluorescence intensity of the FAM label SpA1-1 itself is stable at 7200. When MGO was added, the fluorescence intensity of the system decreased rapidly and underwent 96.3% quenching within 8 min, reaching equilibrium at 700. When the split aptamer probe SpA1-2 was added, the fluorescence intensity induced limited signal recovery, suggesting assembly competence without target induction. Subsequent T-2 toxin introduction triggered SpA1-1 was dissociated from MGO and collaborated with SpA1-2 to recognize T-2 toxin. Therefore, the fluorescence intensity was restored within 3 min and remained at 1300 without increasing. In summary, the optimal detection time determined by the fluorescence kinetic experiment was 11 min.

### 2.3. Performance Validation of Split Aptamer Biosensor Based on MGO

**Figure 4:**
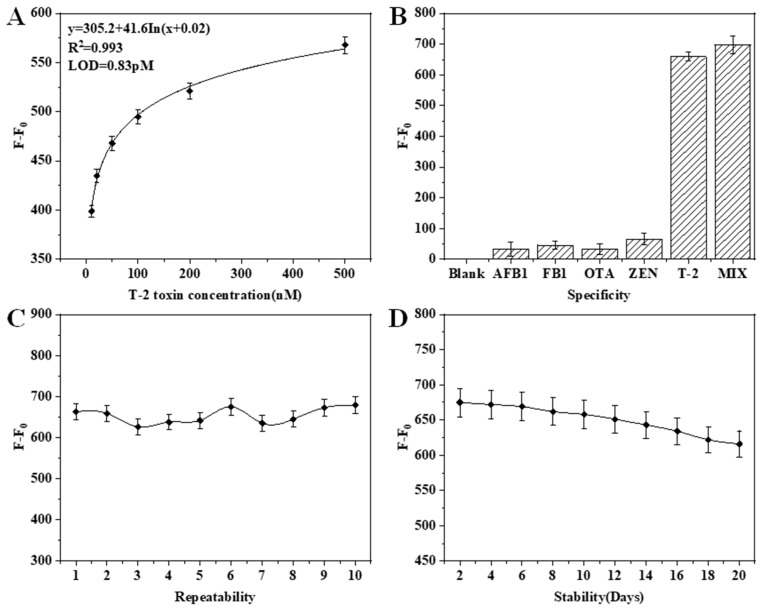
Evaluation of split aptasensor performance: (**A**) Standard curve of T-2 toxin; (**B**) Specificity; (**C**) Repeatability; (**D**) Stability.

Following systematic optimization, the biosensor’s analytical performance was evaluated through four critical metrics: sensitivity, specificity, repeatability, and stability. These were rigorously evaluated and the results are shown in Figure 4. As can be seen from Figure 4A, the split aptamer biosensor has a good logarithmic relationship in the range of 10–500 pM. The regression model y = 305.2 + 41.6 ln(x + 0.02) demonstrated excellent correlation (R^2^ = 0.993), where x represents T-2 toxin concentration and y represents the normalized fluorescence intensity. The detection limit LOD was calculated according to the International Union of Pure and Applied Chemistry (IUPAC) standard (LOD, 3 s/δ) and achieved a sub-picomolar of 0.83 pM, surpassing most reported aptasensors for T-2 toxin (Table 1). Simultaneously, the detection limit was lower than the previous method of using the split aptamer to detect T-2 toxin (0.1 nM, 0.12 nM for FL and FP signals) in our prior study. Figure 4B shows the results of the specificity assessment. As can be seen, the sensor has excellent target selectivity toward T-2 toxin, regardless of whether the interfering toxins AFB1, FB1, OTA, and ZEN are present alone or under co-exposure with T-2 toxin.

Ten independently split aptamer sensors prepared under the same conditions have a high degree of consistency for the fluorescence intensity changes produced by T-2 toxin detection (Figure 4C), with an error of 4.39% ± 0.18. The result shows batch-to-batch consistency and indicates that the constructed detection method has good repeatability. Another 10 split aptamer biosensors were constructed and stored at 4 °C for 20 days, of which 1 was taken every 2 days for the detection of the same concentration of T-2 toxin (200 pM). As can be seen from Figure 4D, the stability of the constructed split aptamer biosensor decreases with the extension of time. However, the stability was good in the first 10 days (error 5.55% ± 0.21). These indicate that the biosensor can maintain good stability during the critical 10-day window.

### 2.4. Detection of T-2 Toxin in Real Samples

To comprehensively evaluate practical applicability of the split aptamer-based biosensor, we selected wheat and beer to represent solid and liquid samples for the recovery tests. As can be seen from Table S2, the recoveries of wheat samples were 86.0–114.2%, and beer samples showed 112.0–129.6% recovery, which revealed that the split aptamer biosensor has good recoveries for the detection of T-2 toxin in solid wheat samples and liquid beer samples. However, we found the difference recovery performance between solid and liquid matrices, especially for beer samples, has a systematic positive bias (>100% recovery across all spiked levels). This may be due to the matrix-enhancement effects. The presence of dissolved organic compounds, including polyphenols and oligosaccharides in beer, may interact with fluorescent reporters or stabilize aptamer-target complexes, inadvertently amplifying the detection signal. Importantly, these results are within the acceptance of the demands, confirming the reliability of the proposed biosensor in complex food systems.

## 3. Materials and Methods

### 3.1. Materials and Instruments

All oligonucleotide sequences used in the experiment were synthesized (HPLC) by Sangon Biotechnology Co., Ltd. (Shanghai, China) (Table S1). T-2 toxin, fumonisin B1 (FB1), zearalenone (ZEN), ochratoxin A (OTA), and aflatoxin B1 (AFB_1_) were purchased from Qingdao Pribolab Biotechnology Co., Ltd. (Qingdao, China). MGO nanosheet solution was purchased from Jiangsu XFNANO Materials Technology Co., Ltd. (Nanjing, China). Binding buffers (BB) were prepared in the laboratory (50 mM Tris-HCl, 5 mM KCl, 100 mM NaCl, and 1 mM MgCl_2_ 6H_2_O, 1% Methanol, pH 7.4), and all other chemical reagents used in the experiment are analytical grade.

Fluorescence signals were quantified using a Synergy H1 multiplex microplate detection system (BioTek, Winooski, VT, USA). All solutions were prepared using ultrapure water produced by the Medium-E400UP Ultrapure Water Vessel (HHitech, Shanghai, China).

### 3.2. Optimization of FAM-SpA1-1 Concentration

Different concentrations (0.5, 1, 2, 5, 10, 20 nM) of FAM-SpA1-1 solutions were subjected to sequential thermal treatments: 5 min at 95 °C using a metal heating block, followed by 10 min incubation on ice, and subsequent equilibration at room temperature for 30 min to facilitate stable spatial structure formation. Then, 200 μL of the FAM-SpA1-1 solutions were transferred to 96-well microplates, and the fluorescence signal was measured by using a multimode microplate reader (excitation wavelength: 485 nm, emission wavelength: 522 nm, the same below). The negative control group without FAM-SpA1-1 solution was set up in the experiment. All experimental groups were analyzed in triplicate, with final fluorescence values calculated by subtracting the mean background signal obtained from control group.

### 3.3. Optimization of Magnetic Graphene Oxide (MGO) Concentration

FAM-SpA1-1 (10 nM) of was heated at 95 °C for 5 min, ice quenching for 10 min, and room temperature equilibration for 30 min to establish stable spatial structure conformation, and then incubated with MGO solutions at different concentrations (10, 20, 40, 60, 80, 100, 120, 140, 160, 180, 200 μg/mL) at room temperature for 30 min, and then 200 μL of the supernatant were subjected to fluorescence quantification after magnetic separation. The negative control group without MGO solution were implemented in parallel. All treatment groups were calculated by subtracting the average background signal from the control groups.

### 3.4. Optimization of SpA1-2 and FAM-SpA1-1 Molar Ratio

FAM-SpA1-1 (10 nM) underwent thermal conditioning at 95 °C for 5 min followed by an ice bath for 10 min and room temperature equilibration for 30 min, then 45 μg/mL MGO solution was added and incubated at room temperature in the dark for 30 min, and then mixed with SpA1-2 solution in different ratios (1:0, 1:0.5, 1:1, 1:1.5, 1:2, 1:2.5, 1:3) for a certain period of time, and the fluorescence intensity was measured after magnetic separation. Negative control (buffer-only) was implemented in parallel. All treatment groups were calculated by subtracting the average background signal from the control groups.

### 3.5. Optimization of Detection Time

Optimization of detection time was investigated through fluorescence kinetic test at room temperature. The experimental protocol was designed as follows: the real-time fluorescence signal of FAM-SpA1-1 (10 nM) was first recorded for 5 min, then 100 μL MGO solution (140 μg/mL) was introduced to initiate the reaction, with the real-time fluorescence signal monitoring continuing for 30 min. Following this, 50 μL SpA1-2 solution was sequentially added to the mixture and the fluorescence signal was recorded for an additional 5 min. Finally, 10 μL T-2 toxin solution was added to the previous mixture and the fluorescence signal was extended for 60 min.

### 3.6. Detection T-2 Toxin Based on Established Methods

Under the above optimized conditions, a detection solution containing FAM-SpA1-1 (10 nM), MGO (140 μg/mL), and SpA1-2 (15 nM) was prepared. Then aliquots of this mixture were incubated with serially diluted T-2 toxin standards at room temperature under light-protected conditions. Following magnetic separation, 200 μL of supernatant from each sample was transferred to a black-walled 96-well microplate for fluorescence quantification. At the same time, parallel negative controls were processed identically. The standard curve was established by plotting ΔF (ΔF = F − F_0_, F was the fluorescence value of the experimental group and F_0_ was the fluorescence value of the control group) against logarithmic T-2 concentrations. All experiments were conducted in triplicate.

### 3.7. Repeatability and Stability Test

To evaluate the practical reliability of the split aptamer-based biosensor, repeatability and stability assessments were conducted as follows:

Repeatability: Ten biosensor units fabricated in the same batch were employed to detect a fixed concentration of T-2 toxin (200 pM) under identical conditions. Fluorescence signals were recorded using Synergy H1 multiplex microplate detection system. Each test included a negative control and triplicate measurements were performed.

Stability: Ten biosensors were stored at 4 °C and tested every 48 h over a 20-day period. At each interval, sensors were exposed to 200 pM T-2 toxins, followed by fluorescence measurement. Triplicate measurements with negative controls were performed per sensor per time point.

### 3.8. Real Samples Analysis

To evaluate the practical applicability of the split aptamer biosensor, the recovery tests were conducted using wheat (solid matrix) and beer (liquid matrix) as representative food samples. The sample was treated based on Khan’s and Ma’s method with minor modification [5,26]. Wheat samples were crushed using a grinder, followed by manual homogenization with a mortar and ground into a powder. A 5.0 g of the powder sample was accurately weighed into a 50 mL transparent centrifuge tube. Subsequently, 25 mL of a 50% (*v*/*v*) methanol solution was added, and the mixture was vortex-mixed for 30 min to facilitate analyte extraction. The extract was subjected to centrifugation at 4 °C under 5000 rpm for 20 min. The supernatant was filtered through a 0.22 μm nylon membrane via vacuum filtration to obtain the blank wheat matrix extract. For the pretreatment of liquid samples, 5 mL aliquot of beer was directly transferred to the centrifuge tube. Following the addition of 25 mL of 50% (*v*/*v*) methanol solution, the mixture underwent vortex agitation for 30 min and equilibration at room temperature for 25 min, and the solution was centrifuged under identical conditions as the wheat samples, and the supernatant was collected after centrifugation and filtered with a 0.22 μm filter as the blank beer matrix. To conduct the recovery test, T-2 toxin with different concentrations (100, 200, 400 pM) were added to the above samples. The recovery rate was calculated based on the following equation:

Recovery rate (%)= Detected concentrationAdded concentration × 100%

## 4. Conclusions

This work presents a groundbreaking dual-signal amplification strategy integrating magnetic target enrichment and split-aptamer cooperativity for ultrasensitive, rapid detection of T-2 toxin in food samples. The synergistic interaction between the two split-aptamer strands effectively minimizes false-positive signals, ensuring high assay reliability. The introduction of magnetic graphene oxide significantly improves separation and enrichment efficiency. The developed biosensor demonstrates excellent performance, achieving a broad linear detection range of 10–500 pM and an exceptionally low limit of detection of 0.83 pM. Notably, the assay is user-friendly, requiring only 15 min for completion. Successful application in detecting T-2 toxin in wheat and beer samples underscores its practical utility. This innovative approach holds significant promise for the highly sensitive and rapid monitoring of T-2 toxin in food safety applications.

## Data Availability

The original contributions presented in this study are included in the article/Supplementary Material. Further inquiries can be directed to the corresponding author.

## Supplementary Materials

The following supporting information can be downloaded at: https://www.mdpi.com/article/10.3390/molecules30132853/s1. Figure S1. Chemical structure of T-2 toxin. Figure S2. UV-Vis scanning of T-2 toxin before and after incubation with magnetic graphene oxide. Table S1. List of split aptamer sequences. Table S2. Recoveries of T-2 toxin in real samples.
