# Supplementary material for: Dual Signal Enhancement by Magnetic Separation and Split Aptamer for Ultrasensitive T-2 Toxin Detection
Source: Molecules. 2025 Jul 4;30(13):2853. doi: 10.3390/molecules30132853 (PMC12251544; doi:10.3390/molecules30132853)
Supplement: Supplementary file 1 [file molecules-30-02853-s001.zip › molecules-3687014-supplementary.pdf]

Supporting Information for

Dual Signal Enhancement by Magnetic Separation and Split Aptamer for Ultrasensitive T-2 Toxin Detection

Ziyi Yan [1], Ping Zhu [1], Chaoyi Zhou [1], Dezhao Kong [1,2] and Hua Ye [1,2,*]

[1] School of Grain Science and Technology, Jiangsu University of Science and Technology, Zhenjiang 212003, China; yanziyi20@hotmail.com (Z.Y.); zhupiiing0605@163.com (P.Z.); 242212188423@stu.just.edu.cn (C.Z.); kdz1011@just.edu.cn (D.K.)

[2] Jiangsu Provincial Engineering Research Center of Grain Bioprocessing, Jiangsu University of Science and Technology, Zhenjiang 212003, China

* Correspondence: yehua_2004@163.com; Tel./Fax: +86-511-84423038

Table of Contents

Figure S1 Chemical structure of T-2 toxin

Figure S2 UV-Vis scanning of T-2 toxin before and after incubation with magnetic graphene oxide

Table S1 List of split aptamer sequences

Table S2 Recoveries of T-2 toxin in real samples

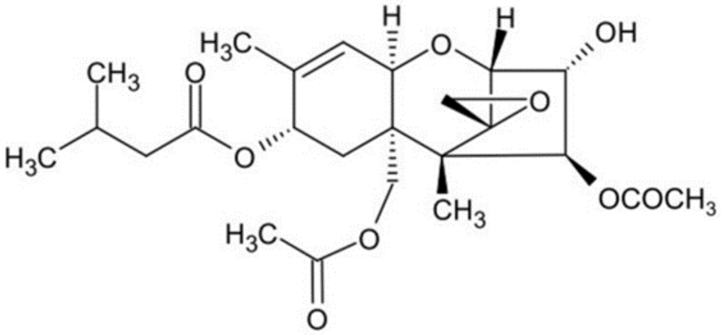

Figure S1 Chemical structure of T-2 toxin

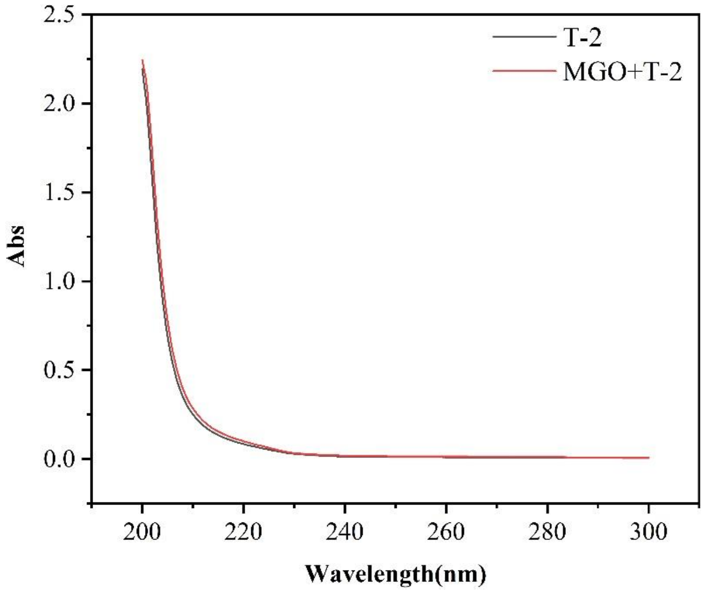

Figure S2 UV-Vis scanning of T-2 toxin before and after incubation with magnetic graphene oxide

Table S1. List of split aptamer sequences

Name	Sequence
FAM-SpA1-1	5'-FAM-CAGCTCAGAAGCTTGATCCTG-3'
SpA1-2	5'-TATATCAAGCATAGAGGTG-3'

Table S2 Recoveries of T-2 toxin in real samples

Samples	Spiked concentration (pM)			Detected concentration (pM)			recovery (%)		
wheat	100	200	400	114.2±10.1	171.7±20.0	378.4±15.2	114.2	86.0	94.6
beer	100	200	400	111.7±8.2	240.7±28.8	518.5±83.8	112.0	120.3	129.6
